# Effect of Temperature and Flow Rate on the Cell-Free Area in the Microfluidic Channel

**DOI:** 10.3390/membranes11020109

**Published:** 2021-02-03

**Authors:** Angeles Ivón Rodríguez-Villarreal, Manuel Carmona-Flores, Jordi Colomer-Farrarons

**Affiliations:** Department of Electronics and Biomedical Engineering, Faculty of Physics, University of Barcelona, 08028 Barcelona, Spain; ivonrodriguez@ub.edu (A.I.R.-V.); m.carmona@ub.edu (M.C.-F.)

**Keywords:** microfluidics, blood plasma separation, cell-free area, microdevice, blood flow

## Abstract

Blood cell manipulation in microdevices is an interesting task for the separation of particles, by their size, density, or to remove them from the buffer, in which they are suspended, for further analysis, and more. This study highlights the cell-free area (CFA) widening based on experimental results of red blood cell (RBC) flow, suspended in a microfluidic device, while temperature and flow rate incrementally modify RBC response within the microflow. Studies of human red blood cell flow, at a concentration of 20%, suspended in its autologous plasma and phosphate-buffered saline (PBS) buffer, were carried out at a wide flow rate, varying between 10 and 230 μL/min and a temperature range of 23 °C to 50 °C. The plotted measures show an increment in a CFA near the channel wall due to cell flow inertia after a constricted channel, which becomes more significant as temperature and flow rate increase. The temperature increment widened the CFA up to three times. In comparison, flow rate increment increased the CFA up to 20 times in PBS and 11 times in plasma.

## 1. Introduction

Portable devices can easily be transported from one point of interest to another. Moreover, another benefit that makes them more attractive is that they can support a wide range of achievable working ranges.

Therefore, with this type of equipment design (while analyzing a sample), one must consider the possibility of handling error and environmental variations. Preliminary analysis of these alterations can help eliminate measurement errors or present advantages for later applications.

The effect of temperature on red blood cells (RBCs) have thoroughly been studied via biotechnologies and other medical applications (for example, how high temperatures, such as 50 °C, do not cause damage and hemolysis (premature RBCs destruction) if exposure is less than 20 min [1]). On the other hand, Weon, and Hwan (2002) found that, after blood samples were exposed at 50 °C for 5 min, there was no change in erythrocyte count, hemoglobin concentration, or mean corpuscular hemoglobin. However, there were changes in erythrocyte morphology after 30 s, as they observed echinocyte apparition in the samples.

In microfluidic blood applications, temperature increments affect the behavior of whole blood in the circulation, decreasing viscosity [2], and increasing cell membrane deformability [3] and plasma protein degradation at temperatures over 43 °C [4].

In D. M. Eckmann’s research [4], RBCs were suspended in different plasma expanders or hemodiluents. Moreover, they were exposed to temperature variations, and it was discovered that the cell suspensions responded differently depending on the solution. The viscosity cell suspension decremented by 26% in hydroxyethyl starch (HES), 23% in its autologous plasma, 21% in 0.9% NaCl, and 14% in 5% albumin solution, when temperature increased from 15 °C to 37 °C.

Other experiments performed with samples from septic patients used dilutions of HES, 0.9% NaCl, and 5% albumin to study rheological changes between them. The dilution of HES showed less erythrocyte deformability and more significant cell aggregation, increasing blood viscosity; meanwhile, albumin diluent resulted in lower blood viscosity even over a wide range of shear rates [5,6].

Nowadays, biomedical experiments and research laboratories use a biocompatible phosphate-buffered saline (PBS), mostly composed of NaCl, to wash and re-suspend the RBCs, eliminating the clogging proteins that could hamper the continuous flow in microchannels. The absence of these proteins also affects the blood cell deformability, representing a change in the cell suspension’s viscosity [7,8].

In addition to temperature, hemodiluents and the fluid’s flow rate variation also impacts blood behavior [9]. Blood has a non-Newtonian (viscosity shear dependents) behavior at shear rates below 150 s^−1^ [4]. At low shear rates, the apparent blood viscosity increases due to the cell sedimentation and the cell aggregation caused by macromolecular plasma proteins (such as fibrinogens) [10,11]. At higher shear rates, there is a disaggregation of cells and an exponential decrement of viscosity. Chien et al. (1990) reported that apparent viscosity decreases from 100 cP to 4 cP as the shear rate increases from 0.01 to 1000 s^−1^ [11]. As the flow rate increases, the cells are elongated by the shear stress and aligned with the flow lines’ direction [12]. At one point, cells arrive at the elongation limit, and the viscosity reaches a plateau. As there are no apparent changes in the viscosity due to the shear rate, the blood flow is considered Newtonian. Skalak et al. (1989) found that blood’s apparent viscosity became constant at shear rates greater than 1000 s^−1^ [13].

The present study analyzes RBCs suspension behaviors at a wide range of temperatures, between 23 °C and 50 °C. Although body temperatures over 42.5 °C are quite rare, this research studies higher temperatures as a supplemental analysis to other published articles, analyzing the effect on blood flow due to cell membrane variations at 50 °C for in vitro studies [1,14] and medical applications.

RBCs are subject to different shear rates in physiological conditions depending on the vessels’ section and diameter. The smallest arteries have a 2 × 10^3^ s^−1^, but in atherosclerotic arteries, it rises to 40 × 10^3^ s^−1^ [15]. In this research, to analyze the impact of a wide range of flow rates on the cell-free area (CFA) width, the RBCs are suspended in PBS and its autologous plasma.

The Fåhræus–Lindqvist effect proved the cells’ tendency to flow around the blood vessel’s center core, leading to a cell-free layer along its wall. This effect changes due to the vessel size or the hematocrit concentration. In this research, CFA can be increased significantly by adding a narrower section to modify the flow pattern behavior.

The channel geometry used comprises a narrow channel to speed-up the cell suspension, flowing downstream into a wider outlet microchannel. The increase in velocity contributes to forming a CFA, a layer of plasma or PBS absent from the cells observed in the exit channel, between the channel’s solid wall and the cells’ flow. It is studied how this measure widens as the temperature or flow rates increase.

RBC aggregation is not taken into consideration for this study. Previous publications reported that red blood cell aggregation appears at low shear rates <10 s^−1^ [16,17], and according to Pop et al. (2002), cells disaggregate from shear rates of 100 to 200 s^−1^ [18].

In the present study, flow was subjected to a high shear rate of 226 s^1^ at 10 μL/min and 5.2 × 10^3^ s^−1^ at 230 μL/min. For this, the flow was passed through a narrower section at shear rates of 4.5 × 10^3^ s^−1^ at 10 μL/min and 104 × 10^3^ s^−1^ at 230 μL/min.

The use of constricted sections to create a wider area free of particles has already been studied as a response to the flow inertia [19]. It has been observed in ovine RBCs [20] and at high concentrations of human cell 16% flow [21].

This research shows the experimental results of analyzing the CFA at a wide range of flow rates (shear rates) and temperatures. Flow rates from 10 to 230 μL/min at 45 °C increase CFA from 15 to 224 μm in plasma and from 20 to 187 μm in plasma. On the other hand, at 27 °C, the CFA increases from 10 to 195 μm in PBS and from 15 to 165 μm in plasma.

Moreover, temperature increment from 23 °C to 50 °C at 100 μL/min increases CFA from 29 to 94 μm in a plasma solution.

### Basic Theory

The ratio between the inertial and viscous fluid forces results in a dimensionless number called Reynolds (Re). When this number remains low, Re < 50 [22], a laminar flow governs in the system, which is a usual microfluidics condition where flow direction, velocity, and other flow properties remain constant over time. Besides, the flow lines flow in parallel with the channel walls where they are confined.

Small particles suspended in the flow tend to remain in the flow line where its mass center is. Nevertheless, in microfluidics, the velocity profile along the channel is parabolic, where the velocity in the center is maximum, and near the wall is nearly zero (no-slip condition). This velocity variation along the transversal channel section causes shear stress between flow lines, maximum near the channel wall, and decreases toward the center, affecting particle behavior.

At higher shear stress, there is more red blood cell membrane elongation [23]. According to Newtonian Law (Equation (1)), shear stress (τ) is the viscosity (η) times the shear rate ($\dot{\gamma }$).

(1) $$\tau =\eta \cdot \dot{\gamma }$$

This analysis defines the shear rate (Equation (2)) as the mean velocity (*u*) divided by the high (*h*) channel.

(2) $$\dot{\gamma }=\frac{du}{dh}$$

Moreover, the flow rate (*Q*) used to inject the flow sample is the volume (*V*) of fluid that passes through a cross-section area (*A*) during a period (*t*). It can also be represented in the function of flow velocity (*v*) (Equation (3)).

(3) $$Q=\;\frac{V}{t}\;\;\;\;\;\;\to \;\;\;\;\;\;Q=\;v\cdot A$$

During the experimental phase, the increment of flow rate increases the shear rate and shear stress [24]. However, within the channel, the parabolic velocity profile establishes a low velocity near the walls, translating it into greater flow resistance.

The fluid resistance ($R_{f})$ for a rectangular microchannel (Equation (4)) is represented by the gradient of pressure (∆*P*) within the channel, the flow rate (*Q*), and the cross-section area (*A*) [25]. In Equation (4), the flow rate is substituted by Equation (3).

(4) $$R_{f}=\;\frac{∆P}{v\cdot A}$$

Moreover, it is well known that there is an inversely proportional relationship between the fluid resistance and the flow rate [26]. The lower the velocity, the greater the resistance to flow. Therefore, a greater force opposes the particles’ displacement by the wall where a CFA is observed compared to the central core with higher particle concentration.

Many research groups have spent time and resources analyzing how cells or particles that flow near the channel wall experience a lifting force that moves them away from it [27,28,29,30]. In terms of blood application, the Fåhræus–Lindqvist effect occurs in small diameter blood vessels of less than 300 μm, where cells migrate to the vessel’s center area, leaving a CFA along the vessel’s wall [31,32], this effect seems to be more perceived as vessels size decreases.

This investigation is based on a narrow channel to recreate this effect and increase the red blood cell’s flow speed. The flow will then flow into an outlet channel to project the CFA into a wider section. The cells’ higher velocity of flowing downstream, following the flow lines, also contributes to wider the plasma section.

## 2. Materials and Methods

### 2.1. Microfluidic Device Design and Fabrication

**Figure 1:**
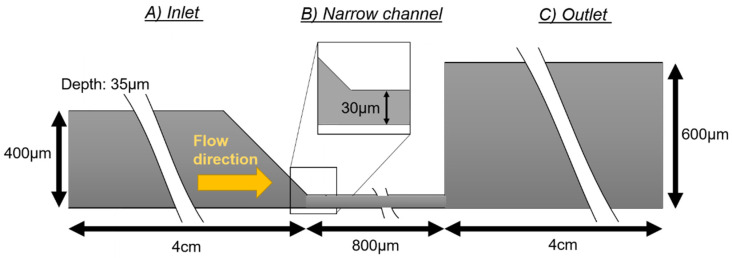
The figure represents the geometry of the microfluidic channel, with a constant depth of 35 μm. The length of the (**A**) inlet section is 4 cm long and 40 μm wide. The (**B**) narrow channel is 30 μm wide and 800 μm long. The (**C**) outlet section is 4 cm long and 600 μm wide.

The geometry of the device consists of a channel with three sections (Figure 1). The first section is the fluid inlet channel (400 μm wide and 4 cm long), which ends in a funnel shape to avoid vortex and bubble formation at the corners and redirect the flow into a narrower channel (Figure 1A). The narrow channel has a width of 30 μm and a length of 800 μm. Its main function is to accelerate the speed of the fluid (Figure 1B) that enters into the outlet channel (600 μm wide and 4 cm long) (Figure 1C). The whole system has a height of 35 μm.

The fabrication technique used is soft lithography. After thoroughly cleaning a glass substrate for the mold fabrication, it was covered with a SU-8 50 resin (Kayaku Advanced Materials, Westborough, MA, USA) poured on it and spun at 4000 rpm for 30 s. The coated substrate was soft-baked for 3 min at 65 °C, and 7 min at 95 °C to evaporate solvents from epoxy.

The channel pattern design was printed on a transparent acetate sheet at 3600 dpi resolution. The mask was placed on the substrate and exposed for 10 s at 22.1 mV/cm² of UV light. The polymerize areas remained attached to the substrate, and then, using hard-bake, increased the cross-linking reaction initialized at the exposure step.

The substrate was dipped in a developer (Kayaku Advanced Materials, Westborough, MA, USA) to remove the non-exposed soluble areas. The remaining structure was the mold for making multiple replicas of the microchannel.

Polydimethylsiloxane (PDMS) (Dow Chemical Company, Madrid, Spain) is a polymer obtained by mixing a base part and a curing agent (10:1). The mixture was placed on a vacuum desiccator to remove all of the air bubbles before pouring it on the pattern mold. It was left at room temperature on a flat surface until it solidified. The PDMS was then peeled off from the mold to obtain the PDMS microfluidic channels.

After bonding the channel and a glass substrate with oxygen plasma, the fluidic connectors were attached to the final prototype.

### 2.2. Experiments Set-up and Sample Preparation

**Figure 2:**
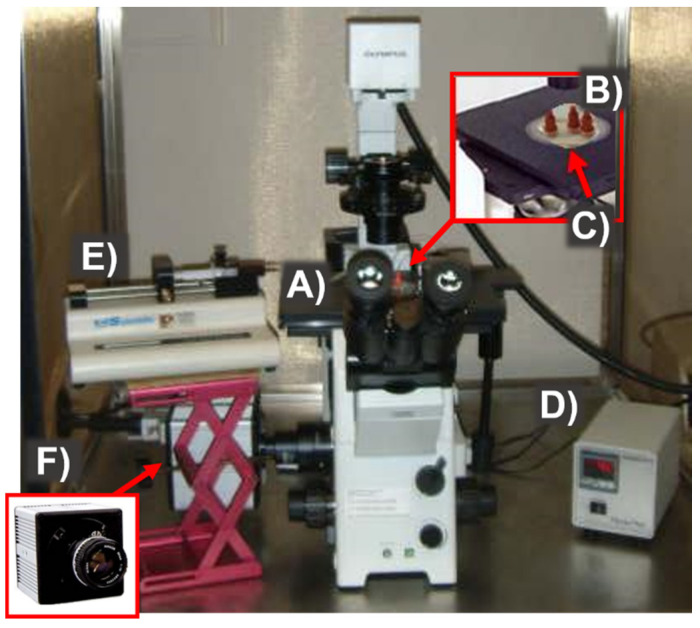
Set-up and instrumentation for experimental procedures. (A) inverted microscope, (B) microfluidic device, (C) thermoplate, (D) thermal device/control, (E) syringe pump, and (F) high-speed camera.

The experimental set-up included a syringe pump (KDS101 CE, KD Scientific, Holliston, MA, USA) to control the inlet flow rates, as well as an inverted microscope (Olympus IX71 from Olympus, London, UK) equipped with a thermal plate (ThermoPlate from Tokai-Hit, Fujinomiya, Japan) to place and heat the PDMS device, and a thermometer to measure the PDMS temperature (ThermoWorks, Inc., Salt Lake City, UT, USA) (Figure 2).

Images and videos were obtained using a Photonics camera (Photron 1024 PCI FASTCAM Camera from Photron, West Wycombe, UK) and recorded at a frame rate between 1000 and 3000 frames per second (fps) and a shutter speed of 1/10,000 s. The CFA distance was measured using the Photron FASTCAM Viewer software.

Blood samples were from anonymous donors and stored at 4 °C for no more than three days after extraction. The whole blood samples were centrifuged three times at 2500 rpm for 4 min to remove the plasma and wash the cells in PBS.

Cells were re-suspended in their autologous plasma, or in PBS, at a concentration of 20%, to study the effect of cell flow suspended in these two solutions at a wide range of temperatures and shear rates.

## 3. Results and Discussion

### 3.1. Flow Rate Simulation and Experiments

Commercial software, based on the Finite Element Method (FEM), was used to simulate flow rate velocities (CoventorWare^®^) along the channel and particle flow lines (ANSYS). The interaction between particles, the particle deformation, such as RBCs, and the use of different buffers, were not considered in any simulation.

For these simulations, the viscosity was assumed constant (Newtonian behavior) regardless of the shear rate. Viscosity was set at 3 cP, a viscosity value known of blood at 20 °C.

**Figure 3:**
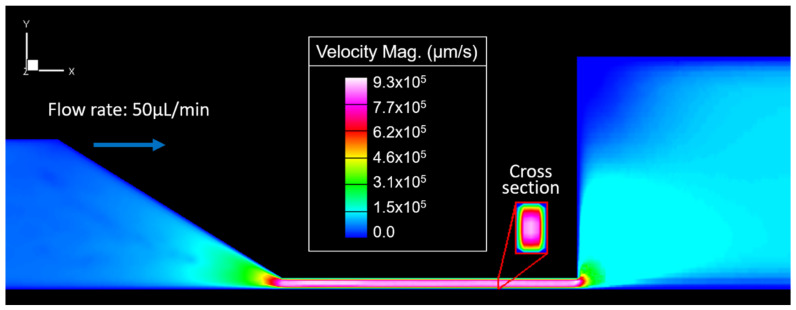
Velocity magnitudes along the channel represented by a color gradient.

Figure 3 shows the flow velocity variations in the microdevice while the fluid was injected at a constant flow rate of 50 μL/min (shear rate of 1130 s^−1^ in the outlet channel). The color gradient shows that velocity near the channel wall is close to zero and that flow increases to the maximum velocity (9.3 × 10^5^ μm/s) in the narrow channel’s central section. Then, velocity decreases as fluid moves away from the narrow channel. It falls more rapidly in the y-direction than in the x-direction. It translates into a lower flow resistance in the x-direction at the outlet channel’s inlet (Equation (4)).

**Figure 4:**
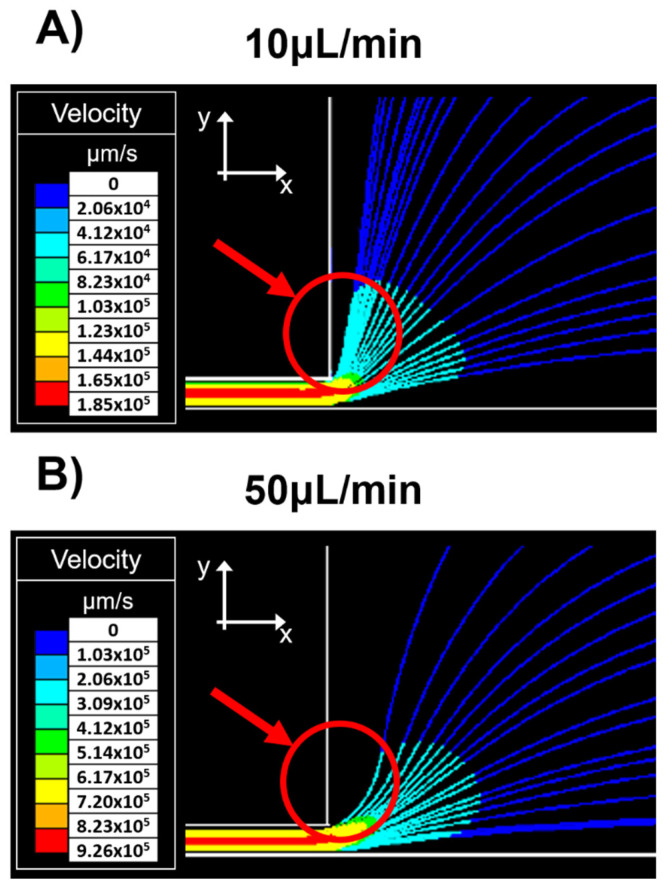
Simulation of tracer particles at the outlet of the narrow channel. (**A**) 10 μL/min and (**B**) 50 μL/min. The color gradient represents particle velocity.

For flow line simulations, tracer particles were placed in the same coordinates at the inlet channel distributed along the cross-section and suspended in a Newtonian fluid. Figure 4 shows the particles’ trajectory just as they exit the narrow channel. At low flow rates, Figure 4A (10 μL/min), particles suspended near the constriction’s upper wall undergo a deflection with a tendency to the y-direction. While at higher flow rates, Figure 4B (50 μL/min) particle deflection has a higher velocity in the inflow x-direction.

**Figure 5:**
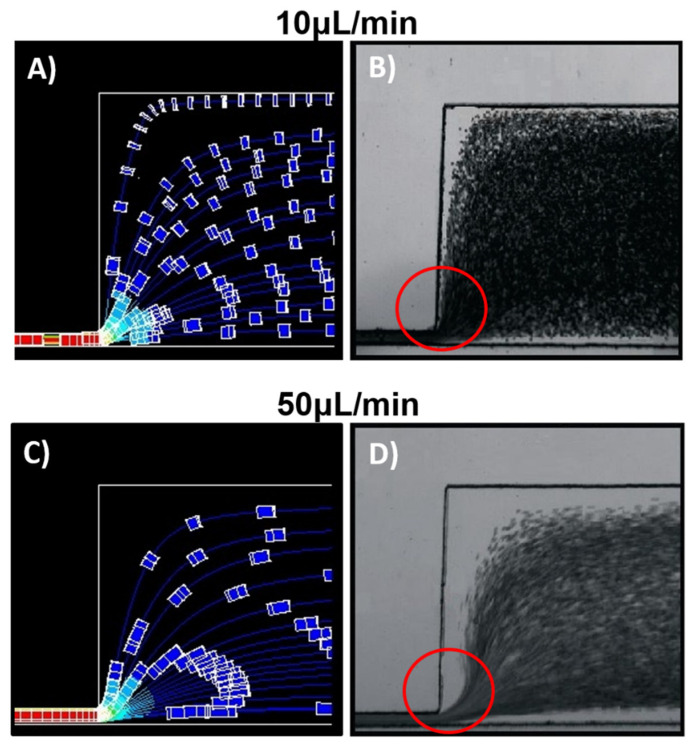
Recorded video images (**B**) (10 μL/min) and (**D**) (50 μL/min) show the phosphate-buffered saline (PBS) cell flow suspension at 27 °C. (**A**) and (**C**) are particle suspension simulations at 10 and 50 μL/min, respectively. The section enclosed in a circle highlights the flow as the red blood cells (RBCs) exit the narrower channel.

Figure 5 shows the simulated results at 10 uL/min (Figure 5A) and 50 uL/min (Figure 5C) of particles suspended in a constant viscous flow. Tracer particles flow rapidly downstream of the channel and move towards the area where the flow velocity is greatest, within the channel’s central core. The particles tend to avoid flows near the walls where the speed is nearly zero, and, therefore, there is a greater resistance to flow.

As the shear rate increases from 10 to 50 μL/min (Figure 5A,C), a CFA appears along the outlet channel walls, which seems wider on the wall opposite the constriction’s outlet. As shown in Figure 4, particles at higher flow rates have a greater velocity component in the x-direction. As particles flow away from the constriction, they slow down (Figure 3), which contributes to get a wider CFA in the opposite wall far from the constriction. The experimental studies of flow rate variations in cell suspensions were carried out at 27 °C and 45 °C, suspended in PBS and plasma. Figure 5B,D show the flow cell response at 10 and 50 μL/min at a 27 °C. The greater the flow rate, the wider the CFA formed along the channel wall.

The suspension was subjected to flow variations from 10 to 230 µL/min to analyze, in more detail, the change in the CFA as the flow rate increases.

**Figure 6:**
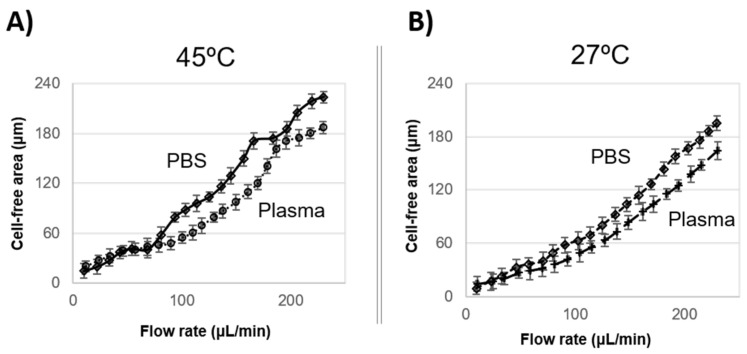
Plot of cell-free area (CFA) responds to flow rate increment at (**A**) 45 °C and (**B**) 27 °C.

The experimental results plotted in Figure 6A, 45 °C, and Figure 6B, 27 °C show a significant increment of the skimmed plasma or PBS results of the flow rate increment.

Analyzing deformable particle suspension in inertial flow at low capillary numbers, Chiara, FL et al. (2020) found that particles are more sensitive to shear rate variations, which is more remarked as particle concentration increases [33]. The interaction between particles increases their shape deformability and the channel’s central core concentration due to particle migration. On the other hand, rigid particles occupied a larger traversal area, decreasing the particles-free area near the channel wall.

Di Carlo et al. (2007) [19] studied the effect of continuous inertial forces in rectangular and curving microchannels and found that channel geometry helps to focus suspended particles inducing inertial migration. Moreover, they analyzed cell-focusing microchannels with diluted cell flow (2% vol/vol) and high flow rates (up to 50 μL/min) and found no significant cell damage at this flow condition.

Faiver et al. (2006) also found that using a contraction–expansion channel had a high impact on the CFA formation [21]. Abay et al. (2020) use a constricted channel to focus 4% of RBCs suspended in PBS [34]. They observed no inertial focusing before the contraction, but the focusing effect was observed due to the narrow section, increasing the velocity.

In this research, 20% of cells suspended in PBS and plasma have an important widening of the CFA after the narrow section. Although the plasma contains the proteins that allow cells to be more deformable, the experiments show that the cells tend to flow downstream in PBS, leading to a wider buffer layer. The authors attribute this effect to the buffer viscosity since it is directly related to flow resistance, and PBS viscosity is 1.8 times lower than plasma, offering less resistance to flow.

Temperature increment from 23 °C to 45 °C reduces PBS viscosity (similar to water) from 0.95 to 0.6 cP, and plasma from 1.75 to 1.09 cP [35].

The experiment shows that, for the same temperature and flow rate conditions, for instance, at 200 μL/min and 27 °C (Figure 6B), the cell flow in PBS reaches a CFA of 168 μm, while in plasma, CFA was 135 μm, 1.25 times smaller than in PBS.

Cell suspension has a velocity about four times greater in the narrow channel compared to the outlet channel. As the cells suspended in PBS come out from the constriction, they face a fluid with lower resistance (than in plasma), and the speeded-up cells flow downstream in the x-direction.

Moreover, as flow rate increases, blood viscosity decreases [36], and cells realign and elongate [17], resulting in an aerodynamic shape that reduces flow resistance.

Moreover, it was observed that the CFA becomes wider at 45 °C compared to those values measured at 27 °C. Therefore, more specific temperature experiments were carried out at a constant flow rate of 100 μL/min and a wide range of temperatures from 23 °C to 50 °C.

### 3.2. Temperature Experiments

Temperature variation was studied at a temperature range between 23 °C and 50 °C and a constant flow rate of 10 μL/min. The temperature set on the thermoplate controller took about 6 min to heat the PDMS device, and a thermometer controlled the device’s temperature.

**Figure 7:**
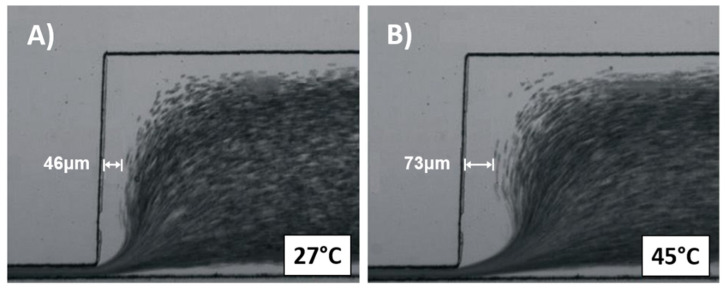
Images of RBCs suspended in plasma at (**A**) 27 °C and (**B**) 45 °C. The measurements are taken in the central section of the outlet channel.

These experiments used cells suspended in plasma. Figure 7A,B show the images taken at 27 °C and 45 °C. The image highlights the CFA distance measured at the center of the channel, at 30 μm from the upper wall. As the temperature increased from Figure 7A, 27 °C, to Figure 7B, 45 °C, the CFA (between channel wall and cell flow profile) increased from 46 to 73 μm, respectively.

**Figure 8:**
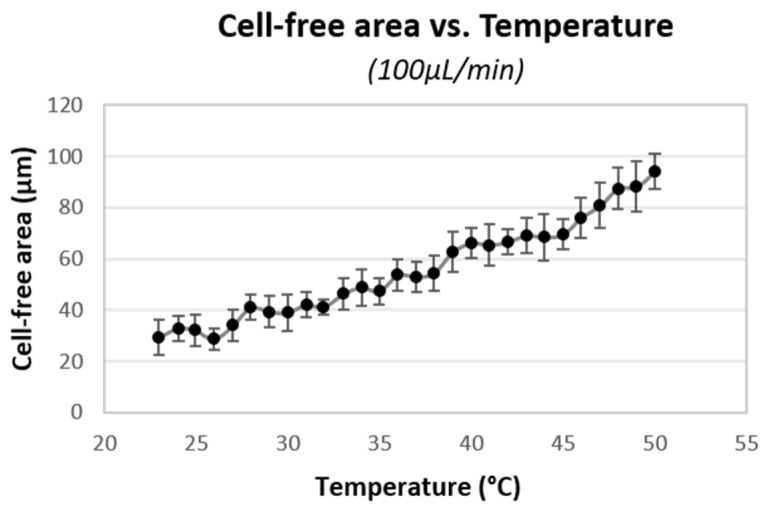
CFA measured at 100 μL/min in a wide range of temperatures, from 23 °C to 50 °C.

Figure 8 plots the CFA’s experimental results between the channel wall and cell flow within the microdevice with the different set temperatures. As temperature increased from 23 °C to 50 °C, the CFA increased 3.2 times, from 30 to 95 μm. Moreover, as the temperature increased, the plasma viscosity decreased 1.6 times, which is a direct decrement in fluid resistance.

This research analyzed the CFA increment in two sections: (1) 24 °C to 37 °C (average body temperature) and (2) 37 °C to 50 °C, with a distance of 14 °C each. In the first section, CFA increased 20 μm, and in the second one, the increment was double, about 42 μm. This CFA widening effect is more significant between 45 °C and 50 °C.

Foo et al. (2006) analyzed blood cells at different flow rates at 23 °C, 37 °C, and 42 °C. They found that, as flow rate increased, cells were aligned, deformed, and elongated, and that higher temperatures implied larger cell membrane deformability. They also found that, at 37 °C or higher, the shear stress needed to elongate cells was minimum [3]. According to this, temperature affects the hemoglobin gelation, the cytoskeleton (spectrin), and the phospholipid bilayer.

Stadler et al. (2008) found that, at 36.9 °C, the internal protein motion (hemoglobin) geometry within blood cells changed [37].

Temperature is a variable that is rarely considered for microdevice design. In addition to the effects that flow rate variations or different buffers can have on the flow, this article proves that small temperature variations greatly impact these biological sample responses within our system. Moreover, the thermal effect can represent an advantage for particle separation or sample manipulation. For this reason, any possible thermal variation involved in microdevice use should be considered in the device design process.

## 4. Conclusions

Studies on cell suspension behavior at different temperatures and flow rates within microfluidic devices can be of interest when it comes to developing new biotechnologies. It is a tool to improve functionality, dismiss possible measurement errors, or add more features to the system.

Within the human body, shear rates vary in blood vessel sizes or circulatory-related diseases. In veins, the shear rate may increase about 10 s^−1^ and 2000 s^−1^ in small arteries. For instance, 1600 s^−1^ in arterioles, 1400 s^−1^ in small arteries, and 200 s^−1^ in the ascending aorta [15]. In pathological conditions, this variable can decrease to 235 ± 341 s^−1^, as in large cerebral aneurysms [38], and increase to 5 × 10^3^ s^−1^ in a stenotic coronary artery; however, according to computational fluid simulations, it can increase up to 200 × 10^3^ s^−1^ [39], depending on the occlusion of the artery.

In this research, blood cell suspensions were subjected to a wide shear rate range, between 10 and 230 μL/min, to study the flow rates’ effect within the cell flow in the microchannel. These values correspond to flow shear rates from 226 to 5.2 × 10^3^ s^−1^ in the wider outlet channel. They were later accelerated when passing through a narrower section, reaching a maximum shear rate of 4.5 × 10^3^ to 104 × 10^3^ s^−1^. In this way, the shear rates that you can find in the human body, for different physiological conditions (both normal and pathological values) have been covered in the same study.

After analyzing flow simulations, it was observed that particles at higher speed tend to flow in the x-direction away from the constriction, resulting in a larger CFA. Blood cell deformability should play a binding effect, to take into account when to analyze blood samples, but in this case, the simulation was considered an approximation of their behavior.

It is important to remember that higher velocities imply a higher shear rate between the flow lines, resulting in changes in viscosity values [40,41] of non-Newtonian fluids (e.g., blood). This fact was not taken into consideration, as the viscosity remained constant during the simulations. The particles’ displacement is then related to their inertia increment within the flow, and not to viscosity variations induced by the flow lines’ shear.

Moreover, before the experiments, blood samples were treated with anticoagulants; therefore, the effect of arterial occlusions, observed at higher shear rates due to platelet aggregation, was not observed in this research [42].

During the experiments, as the flow rate increased, the cell-free area increased in width about 9 times when cells were suspended in plasma and 11 times in PBS, both at 27 °C. Moreover, at 45 °C, the width increased up to 15 times in their autologous plasma, and 19 times in PBS. The use of a narrow section increased the CFA, an effect that was also observed in pathological conditions. Vahidkhah et al. (2016) studied the flow through stenosed microvessels. They found that the CFA decreases significantly in the constricted section but increases as the flow moves away [43]. The variations observed in the CFA in this research due to flow rate changes could also appear in the human body due to fluctuating pulsatile blood flow in the microcirculation.

The experimental results proved that, besides using higher shear rates, the CFA could become wider using a low-viscosity buffer or increasing the flow temperature, reducing the flow resistance.

Although the normothermia is about 37 °C, under abnormal conditions, the human body can be exposed to hypothermia (core temperature under 35 °C), and hyperthermia (over 40.5 °C). Nevertheless, there are extreme conditions when the body core temperature drops to 25 °C or less where asystole occurs, and other health disorders begin to appear [44]. On the other hand, it was found that at 49 °C, cells might die by apoptosis and due to necrosis (at a higher temperature). However, earlier, between 40 °C and 41 °C, an increase of cerebral blood flow may occur, followed by an abrupt flow decrease and culminating in irreversible neuronal damage [44].

Moreover, there are several publications that analyze blood response to even higher temperature variations. The analysis of blood damage after being exposed at higher temperatures of 50 °C for less than 20 min indicated no significant damage to the samples. Later, it was found that, at these temperatures, some cells exposed for 3 s presented morphological changes, becoming echinocytes.

The effect of small temperature variations can lead to severe damage to the human body. This research exposed the blood samples at temperatures between 23 °C and 50 °C for less than 30 s and sped them up by the narrow channel. The use of the wide temperature range covers the entire possible conditions that the circulatory system could experience.

As a result, it was observed that the CFA achieved after the constriction was about three times wider when the temperature increased from 23 °C to 50 °C. The analysis showed that the CFA increased 20 μm, from 24 °C to 37 °C (body temperature), but increased twice, 41 μm, from 37 °C to 50 °C. The effect was more significant between 45 °C and 50 °C. According to other publications, this is related to hemoglobin alterations and cell cytoskeleton modifications [3,37].

Although many studies analyze the cellular effect of temperature in vitro or the triggered impact within the human body, the cell flow pattern in microcapillaries, due to temperature-related disorders, continues to be a study variable to be discovered. The increase of the cell-free area after cell flow is exposed to a higher temperature for half a minute was significant, but more studies may be necessary to clearly understand cell flow at high temperatures under a microfluidic device.

This study is an approach to know the RBC flow effect in medical applications from the hemodynamic perspective. For instance, to understand the circulatory system, abnormal flow conditions as arterial occlusions, and cell-flow pattern behavior, and consider these flow fluctuations for capillaries in organ-on-chip development.
